# Molecular attachment to a microscope tip: inelastic tunneling, Kondo screening, and thermopower

**DOI:** 10.3762/bjnano.10.124

**Published:** 2019-06-19

**Authors:** Rouzhaji Tuerhong, Mauro Boero, Jean-Pierre Bucher

**Affiliations:** 1 Université de Strasbourg, IPCMS UMR 70504, 67034 Strasbourg, France

**Keywords:** inelastic electron tunneling, molecular quantum dot, Kondo physics, single molecule, thermopower, tunnel junction

## Abstract

The vibrational excitation related transport properties of a manganese phthalocyanine molecule suspended between the tip of a scanning tunneling microsope (STM) and a surface are investigated by combining the local manipulation capabilities of the STM with inelastic electron tunneling spectroscopy. By attachment of the molecule to the probe tip, the intrinsic physical properties similar to those exhibited by a free standing molecule become accessible. This technique allows one to study locally the magnetic properties, as well as other elementary excitations and their mutual interaction. In particular a clear correlation is observed between the Kondo resonance and the vibrations with a strong incidence of the Kondo correlation on the thermopower measured across the single-molecule junction.

## Introduction

Scanning tunneling microscopy (STM) has the capability to detect the electron transport through a molecule not only adsorbed on a surface, but also when it is attached to the probe tip itself [1–4]. The sharp tip of the STM is then used to lift a single molecule from the surface in order to efficiently minimize the influence of the substrate on the molecule. In this way, the intrinsic physical properties of the molecule become accessible.

In this context, inelastic electron tunneling spectroscopy (IETS) based on scanning tunneling spectroscopy (STS) has proven to be a powerful technique to investigate and identify molecular objects and their interactions with the environment. The technique allows one to study, with atomic resolution, the coupling of the tunneling electrons to other elementary excitations such as vibrations, plasmons and spins [5–8]. Furthermore, a molecular junction in STM can serve as a reliable and controllable model system for the study of a single molecule in a way similar to the mechanical break junctions [9–12], but with a much better control of the location, number and nature of the molecules.

Transition-metal phthalocyanines with a magnetic center represent a family of simple and robust molecules well suited to study tunable magnetic interactions on surfaces [13–21]. Low-temperature STM/STS is an ideal tool to study the Kondo effect, which manifests itself by a sharp zero-bias resonance in the conductance spectrum of a localized moment on a conducting substrate, due to the coherent spin-flip scattering between the localized spin and the conduction electrons of the substrate. Manganese phthalocyanine (MnPc) molecules adsorbed on noble-metal surfaces have been studied quite extensively by STM and Kondo behavior of MnPc is well documented [19–21]. However the electron transport through a single MnPc molecule suspended in a tunneling junction of an STM has not been studied yet. Here we describe an approach that allows for achieving, in a controlled manner, such a configuration and to analyze in detail how the Kondo correlation affects the IETS and yieds the non-linear thermopower accross the leads.

## Results and Discussion

### Molecule transfer and imaging

**Figure 1:**
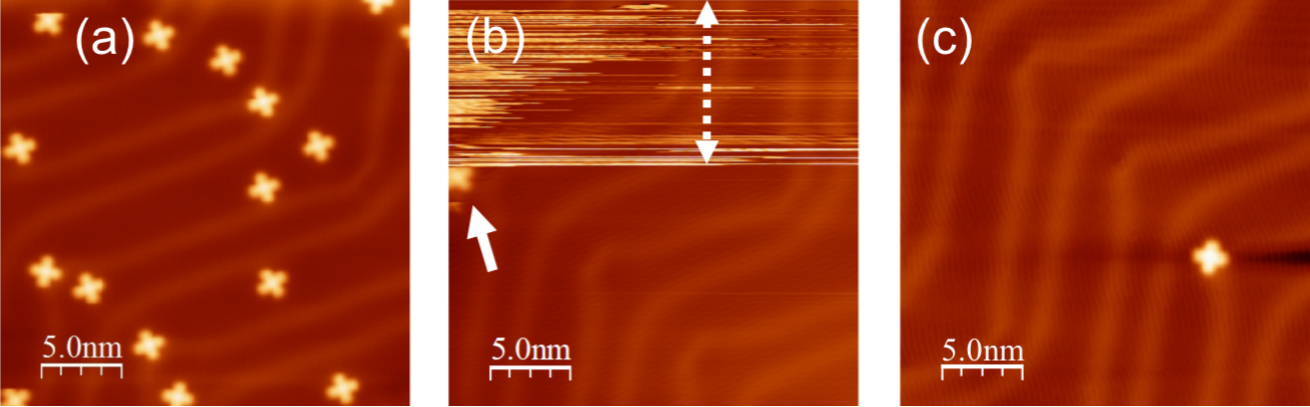
(a) STM image of MnPc molecules on the Au(111) surface (0.3 nA, −0.25 V). (b) STM image of the bare Au(111) surface taken by the STM tip with (upper part) and without (lower part) a MnPc molecule attached to it (0.1 nA, −0.25 V). A single MnPc molecule dropped from the tip during the scanning process (152.5 nm/s) at the position indicated by the arrow. (c) STM image of the MnPc molecule deposited in (b), (0.3 nA, −0.25 V).

Figure 1a shows a topographic STM image of MnPc molecules on Au(111) taken with a Au-covered W tip. As seen clearly from the STM image, the MnPc molecules adsorb face-on. The bright protrusion at the center of MnPc is due to the Mn d-orbital states near the Fermi level [21]. By approaching the STM tip towards a molecule on the Au(111) surface, it is possible to transfer a single MnPc molecule from the surface to the tip apex of the STM. Then the presence of a molecule on the tip can be directly confirmed by the reverse transfer process, i.e., by applying a voltage pulse on the tip or by scanning a clean surface area with this molecule-terminated tip until the molecule drops back from the STM tip to the surface. Since the voltage pulse applied to a phthalocyanine molecule may induce a chemical modification of the molecule through dehydrogenation [13,15], only the second method is used in this work.

**Figure 2:**
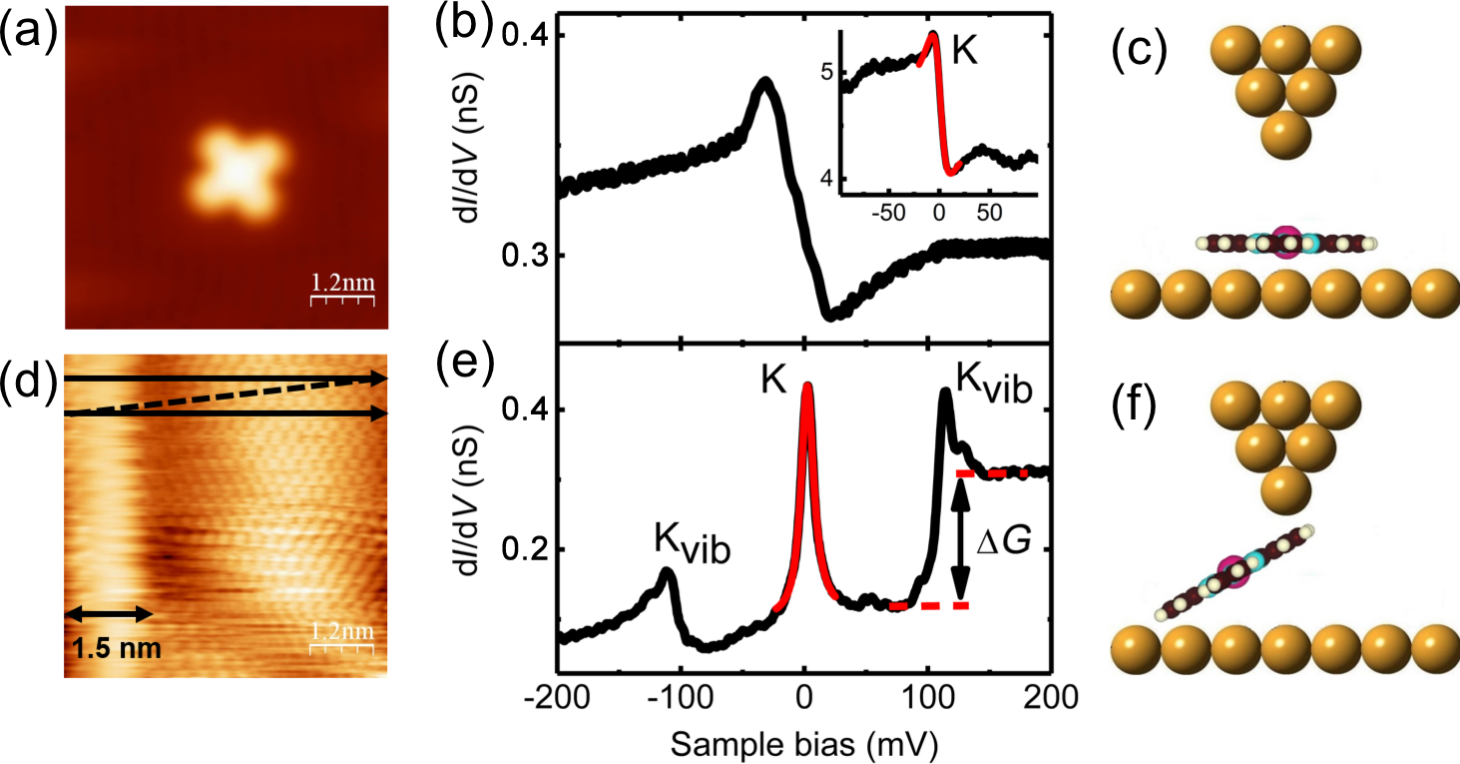
(a) STM image (0.3 nA, −0.3 V). (b) d*I*/d*V* spectrum of a flat-lying MnPc molecule on the Au(111) surface (0.01 nA, −0.3 V). The inset shows a high-resolution spectrum (0.1 nA, −0.1 V) taken with a modulation of 1 mV (rms). The red curve is a Fano line fitted to the Kondo resonance labeled with K. (c) Schematic view of the STM junction for (b). (d) STM image of the bare Au(111) surface with the MnPc-terminated tip (0.1 nA, −0.3 V). The black arrows indicate the raster-scan direction during the STM imaging process. The scanning speed of the MnPc-terminated MnPc tip over the bare Au(111) surface is 19 nm/s, much slower than the one of 76 nm/s in (a). The black double arrow at the bottom-left indicates the width of the bright vertical stripe. (e) d*I*/d*V* spectrum obtained with the MnPc-terminated tip over the bare Au(111) surface (0.1 nA, −0.25 V). The zero-bias Kondo resonance, the vibration-mediated Kondo resonances and the inelastic step in (e) are labeled K, K_vib_, and Δ*G*, respectively. (f) Schematic view of the STM junction for (e).

To pick up the molecule, the tip is approached above the ligand moiety. The ligand moiety is the most favorable site of binding between the phthalocyanine molecule and the sharp metallic electrode as confirmed by calculations [12]. We found that approching the tip above the center of the molecule only leads to a rotation of the molecule around its center whereas the molecule stays on the surface. No current trace was recorded as a function of the tip height, but the tip retraction after molecule attachment is estimated to be about 2 Å. At this stage, the MnPc molecule has been only partially lifted from the surface as anticipated from the STM image of Figure 2d and indicated schematically in Figure 2f (see below).

Because the molecule–tip interaction is stronger than the molecule–surface interaction, it is possible to scan the bare Au(111) surface, with a MnPc-terminated tip. The STM image, however, looks quite noisy as shown in the upper part of Figure 1b. By increasing the scanning speed to 152.5 nm/s, well above the value used for the imaging, the molecule that is initially attached to the tip can be intentionaly dropped to the surface. After dropping the molecule at an elbow site of the Au(111) reconstruction, indicated by the arrow in Figure 1b, the STM image improves significantly and the surface reconstruction of Au(111) becomes clearly visible. It should be emphasized that only one molecule is deposited from the STM tip on the surface as shown in the Figure 1c. Additionallly, the MnPc molecule deposited from the STM tip exhibits the characteristic topographic and spectroscopic features of MnPc adsorbed on Au(111), confirming that this process does not damage the MnPc molecule initially transferred to the tip apex. Furthermore the reversible molecule transfer process between the tip and the surface indicates that the MnPc molecule is attached to the STM tip apex and not to any other site on the tip.

### Vibration-mediated electron transport in a molecular junction

In the following, the electron transport through a MnPc molecule suspended in the gap of the STM is investigated by probing the differential conductance through the junction. The STS spectrum of a MnPc molecule flat-lying on Au(111) is taken with a bare metallic tip and used as a reference spectrum to be compared with the STS result obtained with a MnPc-terminated tip. After attachment of a single MnPc molecule to the tip apex, an STM image of the Au(111) is first taken with this tip (Figure 2d). This image shows a characteristic signature of the presence of MnPc in the STM gap, namely a fuzzy vertical stripe, about 1.5 nm wide, on the left-hand side of the STM image. The width of this stripe corresponds to the size of a single MnPc molecule and originates from the image formation with a molecule-terminated tip one end of which is dragged on the surface during the horizontal line scans as shown by the arrows in Figure 2d. As a result, the atomic resolution of the Au(111) surface by means of the MnPc-terminated tip is obtained only on the right-hand side of Figure 2d.

The electron transport through a single MnPc molecule that is partially lifted from surface (Figure 2f), is further investigated by conducting high-resolution inelastic electron tunneling spectroscopy (IETS) close to the Fermi level. The differential conductance (d*I*/d*V*) spectrum (Figure 2e) reveals a prominent sharp peak close to the zero-bias voltage and two side peaks corresponding to step-like increases in the d*I*/d*V* signal (labeled Δ*G*) at threshold voltages |*V*_th_| = 110 ± 5 meV. This is a typical signature for an inelastic electron tunneling process involving the excitation of molecular vibration modes [5,22]. The IETS features are asymmetric in intensity with respect to the Fermi energy and will be discussed shortly at the end of this section.

There are significant changes in the d*I*/d*V* spectrum between the flat-lying molecule on Au(111) (inset of Figure 2b) and the MnPc molecule attached to the tip (Figure 2e). Lets recall that the first spectrum has been acquired by tunneling into the Mn atom of the molecule adsorbed on the surface (see inset of Figure 2c) whereas the second one has been taken with the molecule attached to the tip by its ligand moiety (see Figure 2f). Therefore, the spectra have been aquired with different set points and the conductances cannot be compared directly. However, it is clear that the d*I*/d*V* spectrum of Figure 2e when the MnPc molecule is suspended in the gap, originates from the interaction of the injected electrons with the various molecular excitations during the tunneling process, not present in Figure 2b. Most importantly, the two IETS side peaks and the conductance step observed for MnPc on the tip are absent when the MnPc molecule is lying on the Au(111) surface.

The feature at zero bias in the spectrum of the flat-lying MnPc molecule of Figure 2b can be attributed to the Kondo resonance arising from the exchange interaction between the MnPc molecular spin and the itinerant conduction electrons of the Au(111) substrate [21]. The red curve in the inset of Figure 2b is a fit to the step-like feature at *E*_F_ by means of a single Fano function with a negative asymmetry factor of *q* = −1.2 and a Kondo temperature of *T*_K_ = 56 ± 4 K. This value is consistent with the Kondo temperature reported in previous STM experiments for this system [20]. The Kondo temperature has been extracted from the fit and corrected for temperature and modulation broadening according to [23]. The Kondo origin of the sharp zero-bias resonance for the on-surface MnPc has been ascertained in a previous work by measuring the Kondo peak variation as a function of temperature and by showing that it fits the Fermi liquid theory [21].

The sharp zero-bias peak in the d*I*/d*V* spectrum of a single MnPc molecule attached to the Au-covered probe tip in Figure 2f, shows that the molecule preserves the spin state of the Mn ion similar to MnPc on Au(111) and Ag(111) surfaces [19–21]. Therefore, the localized spin moment of the molecule might be screened as well by the itinerant conduction electrons of the Au tip. This correlated many-body state leads to an enhanced conductance at zero bias. As before, the sharp feature at *E*_F_ is fitted by means of a single Fano function (red curve in Figure 2e) leading to a positive asymmetry factor of *q* = 22.8 and a Kondo temperature of *T*_K_ = 47 ± 3 K. The Kondo temperature of the MnPc molecule suspended in the STM junction is found to be only slightly lower than the one for on-surface MnPc molecules.

Even though both the tip and the surface are chemically identical (see Experimental section), the MnPc molecule attached to the tip is subjected to a different electronic-state hybridization than an on-surface molecule, and hence a different Kondo behavior is expected. In this respect, the analysis of the fitting parameters of the Fano resonance provides additional clues. The increase in the Fano factor, *q*, from −1.2 for the on-surface molecule to 22.8 for the suspended molecule clearly indicates different electron-tunneling conditions. Since the suspended molecule is attached to the tip, the probability of electron tunneling from the STM tip into the molecule is increased, while tunneling into the bulk continuum of states is significantly decreased, yielding an increase of the *q* value [24]. In the case of the suspended MnPc molecule of Figure 2e, the two side peaks at finite bias accompanied by the zero-bias Kondo peak can be ascribed to the vibration-assisted Kondo resonance for which the excess energy of the electrons due to a finite voltage is compensated by activating a vibration mode [12,25–27]. In addition, based on the comparison with the previously reported STM-IETS of FePc/Ag(111) [28] and theoretically calculated vibration modes of transition-metal phthalocyanines [29–30], the two steps at |*V*_th_| = 110 ± 5 mV are easily assigned to the excitation of the stretching of the Mn–N_iso_ bonds of the MnPc molecule.

The conductance step Δ*G* at the positive threshold voltage of 110 ± 5 mV, corresponds to an increase in the junction conductance of about 200% (see Figure 2e). Such a large conductance increment supports the strong electron–vibration interaction of the MnPc molecule suspended in the STM gap, similar to the one reported for CO_2_ molecule in the platinum break junction [31–32]. Furthermore, the spectral intensity of the MnPc junction shows a strong asymmetry with the polarity of the junction. The asymmetry is difficult to explain by simple means. However, we noticed that for the large-gap resistance (larger tip–sample distances) the effect progressively washes out, see the spectra of Figure 4a below. As a matter of fact, in our configuration, the tip–molecule and the molecule–sample contacts are not equivalent for small gaps, leading to strong effects for partially lifted molecules, as described in Figure 2f. A molecular rearrangement due to new geometrical boundary conditions is thus expected for larger gaps where the tunneling barrier asymmetry at the STM–molecule junction becomes less critical. This asymmetry in the spectrum may suggest that two different vibration-assisted electron-transfer processes apparently compete, one involving a vibration-mediated Kondo effect, and the other, an inelastic tunneling through excitation of molecular vibration modes.

### Parameter-dependent transport in a molecular junction

In order to gain additional information about the kinetics of carriers not contained in the current–voltage characteristics we want to evaluate the impact of the zero-bias peak on the thermopower accross the molecular junction. Interesting thermoelectric effects in the presence of Kondo correlations were anticipated quite early [33]. In particular, sign changes of the thermopower at low temperatures when entering the Kondo regime. Whereas most experiments to date have been performed on solid-state quantum-dot devices, those on molecular junctions and in the presence of Kondo correlations are more recent [34–35].

**Figure 3:**
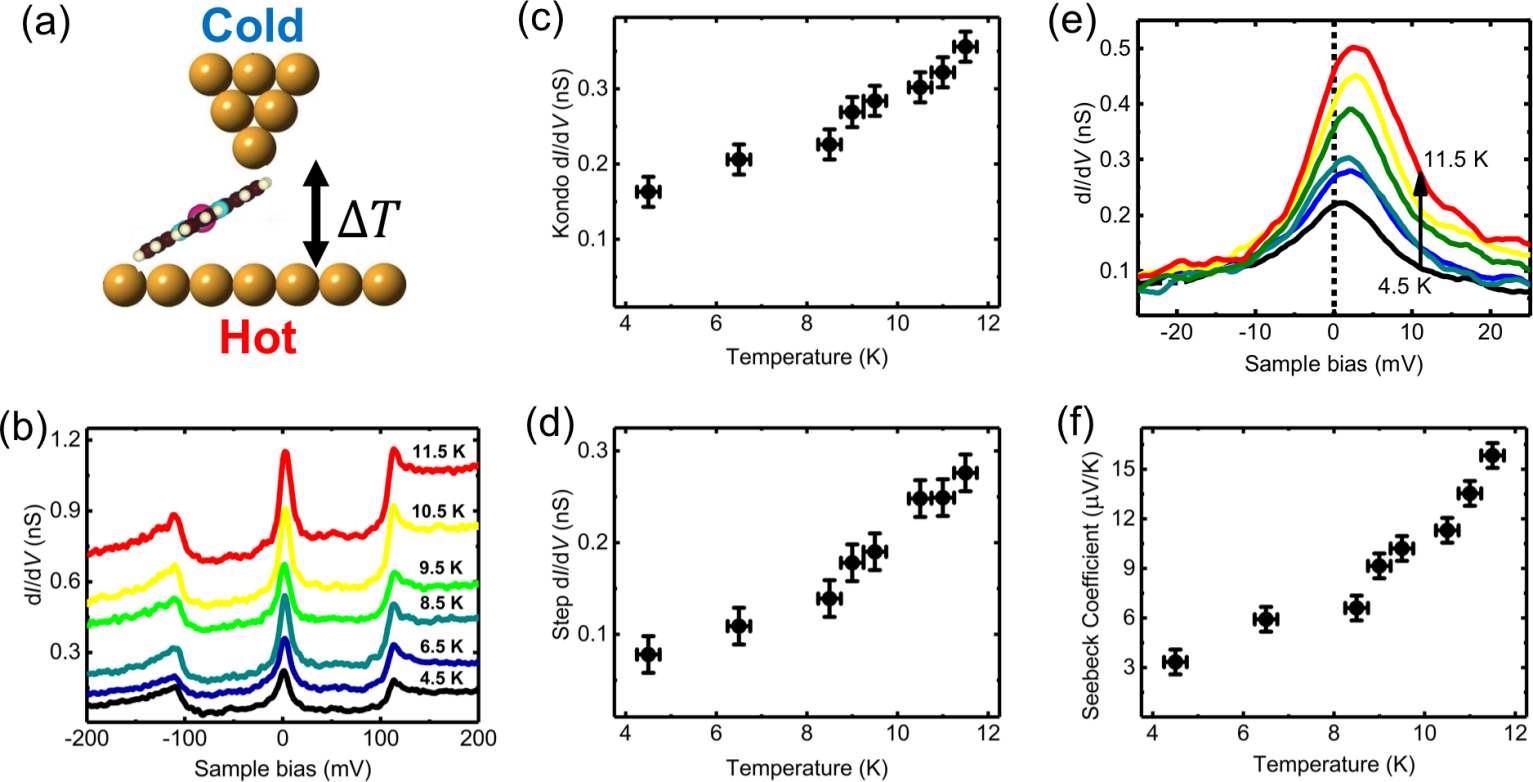
(a) Schematic view of the STM junction; the tip temperature *T*_t_ = 4.5 K is kept constant during the experiment. (b) d*I*/d*V* spectra of the MnPc junction at different sample temperatures *T*_s_, shifted vertically for clarity. The feedback loop has been opened at 0.1 nA, −0.30 V. (c) Maximum conductance of the zero-bias peak as a function of the sample temperature *T*_s_ . (d) Conductance step at positive bias as a function of the sample temperature *T*_s_. (e) Close-up of the d*I*/d*V* spectra showing the zero-bias values (vertical doted line). (f) Seebeck coefficient as a function of the sample temperature *T*_s_ extracted from the value measured in (e).

Here, we analyzed the influence of a temperature gradient on the conductance spectrum of the MnPc molecule in the STM junction, as shown schematically in Figure 3a. Prior to the experiment, the whole STM stage has been cooled to 4.5 K for several days, then the sample is heated-up over a short period of time of a few minutes. At the same time, due to the thermal inertia of the STM stage the tip temperature is assumed constant and equal to *T*_t_ = 4.5 K (see Experimental section). During the step-by-step temperature increase, the feedback loop was “on” (0.2 nA, −0.3V) to prevent accidental loss of the MnPc molecular junction. As soon as the stable sample temperature was reached, the feedback loop was opened (0.1 nA, −0.3 V) and the d*I*/d*V* spectra were acquired at the stabilized sample temperature. Figure 3b presents the d*I*/d*V* spectra measured accross the molecular junction when the substrate is warmed up from its base temperature of *T*_s_ = 4.5 ± 0.1 K to *T*_s_ = 11.5 ± 1.0 K. The resulting temperature variations of the central zero-bias peak and the finite-bias conductance step are displayed in Figure 3c and Figure 3d, respectively. Remarkably, the conductance of both central peak and positive bias step increase as a function of the temperature gradient while the Kondo temperature measured for the different spectra of Figure 3b remains constant and equal to *T*_K_ = 56 ± 3 K. This result is not surprising since 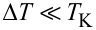 is too small to produce any smearing of the Kondo resonance [36].

The Seebeck coefficient *S* can be calculated from the d*I*/d*V* data as a function of the temperature, obtained at constant height with an open feedback loop [37]:

[1] $$S=\frac{\pi^{2}k_{\text{B}}{}^{2}}{6e^{2}}\frac{\Sigma }{\sigma }\frac{\left(T_{\text{s}}^{2}-T_{\text{t}}^{2}\right)}{\Delta T}\;,$$

where σ(V) is the differential conductance and Σ(V) is its derivative. The conductance is given in Figure 3e, in a small interval around the Fermi energy. The calculation by means of Equation 1 yieds the non-linear Seebeck coefficient *S* that is found to increase with Δ*T*, as shown in Figure 3f. For example it is found that *S* doubles from Δ*T* = 2.0 K to Δ*T* = 6.0 K for which it reaches a value of 13.6 ± 3 μV/K. This value is comparable to the largest values reported for single-molecule junctions [34].

**Figure 4:**
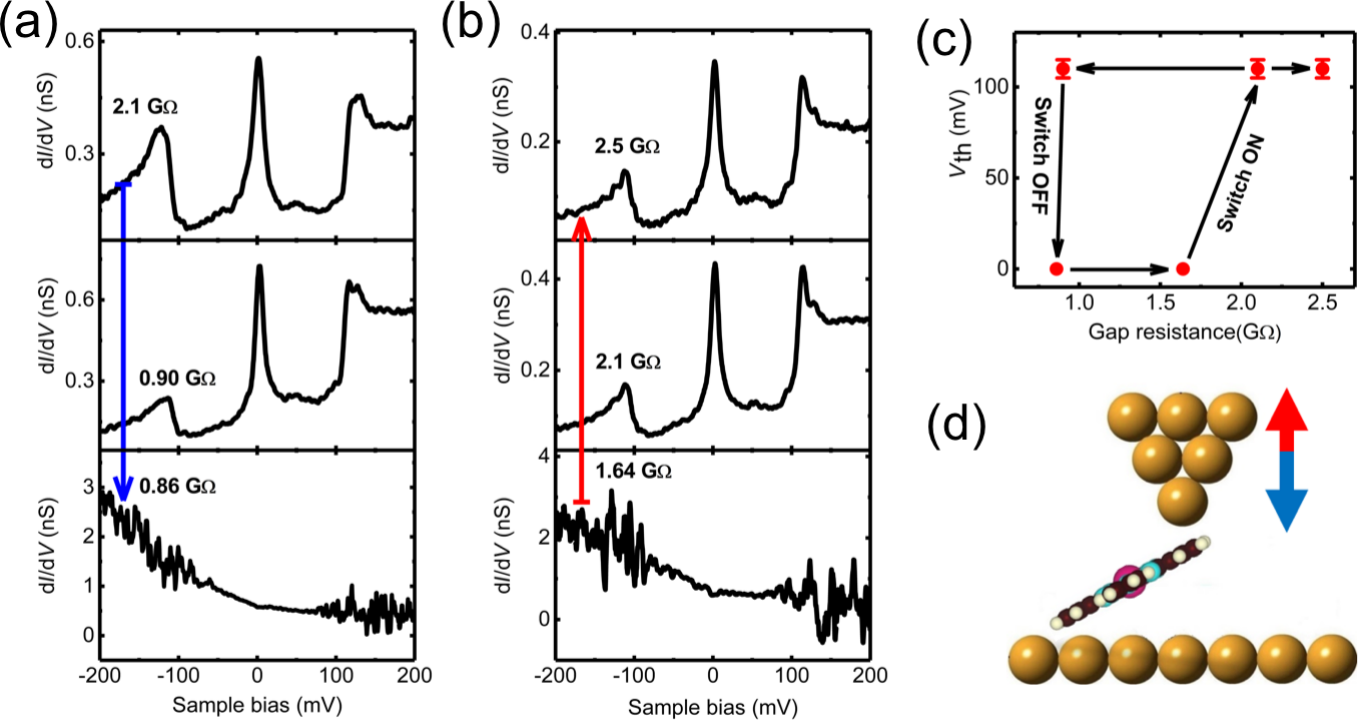
d*I*/d*V* spectra of the MnPc molecular junction with (a) decreasing and then (b) increasing gap resistance, respectively. The gap resistance is controlled by adjusting the sample bias voltage from −250 over −108 to −103 mV, first, and then from −197 over −250 to −300 mV, while the tunneling current is kept constant at 0.12 nA. (c) Variation of the satellite peak threshold voltage as a function of the gap resistance in (a) and (b). (d) Schematic view of the molecular junction. The blue and red arrows indicate decreasing and increasing gap resistance or tip height with respect to the surface.

In order to investigate further the relation between the zero-bias peak and the satellite peaks, we focused on the evolution of differential conductance spectra as a function of the tunneling gap resistance, i.e., tip–sample distance. Figure 4a and Figure 4b present a series of d*I*/d*V* spectra as a function of the gap resistance.

As can be seen in Figure 4a, the marked steps at ±(110 ± 5) mV in the spectra still reveal an efficient coupling between the Mn–N_iso_ stretching mode and the Mn d-orbital, which bears the unpaired spin. The evolution of the spectral asymmetry discussed above is also quite noticeable, being much less pronounced at a gap resistance of 2.1 GΩ than at 0.9 GΩ. Interestingly, both the zero-bias and IETS side peaks are completely and simultaneously quenched when the gap resistance is slightly decreased from 0.90 to 0.86 GΩ. The disappearance of the Kondo and IETS side peaks in the d*I*/d*V* spectrum is most probably due to a change in the configuration of MnPc molecule in the junction between the tip and surface. The loss of IETS steps in the d*I*/d*V* as a function of the gap resistance in Figure 4a, is a very sharp effect and corresponds to a sudden configurational change of the molecule in the STM gap. Simultaneously, a telegraphic noise appears at bias voltages larger than the side-peak threshold, which is often regarded as the signature of an unstable tunneling junction. As shown in Figure 4b, when the resistance is increased again above 1.64 GΩ, both the Kondo resonance and the two side peaks at ±(110 ± 5) mV appear again. Further increasing the gap resistance, does not impact significantly the zero-bias Kondo peak and the side peaks anymore. These results are summarized in Figure 4c, which clearly evidences the hysteretic behavior. The IETS step energies do not change if the loop is repeated again since they are determined by the vibrational modes of the molecule. However, a different asymmetry in intensity of the spectra may appear for different tips.

Interestingly, when the gap resistance is decreased from 2.1 to 0.90 GΩ (blue arrow in Figure 4d), the Kondo temperature *T*_K_ decreases from 60 to 47 K. Following the hysteresis, when the gap resistance is increased from 2.1 to 2.5 GΩ (red arrow in Figure 4d), the Kondo temperature *T*_K_ also increases from 47 to 56 K. This behavior again suggests that the variation of Kondo temperature is due to a change in the tip–sample distance that modifies slightly the configuration of the molecule in the gap. As we can learn from the evolution of the d*I*/d*V* spectra in Figure 4a and Figure 4b, the side peaks always appear as satellite of the zero-bias Kondo peak and are not detected in its absence. This further supports the idea that the side peaks are related to both vibration activation and Kondo transport

## Conclusion

We have described an approach that allows one to achieve, in a controlled way, a configuration where a MnPc molecule is attached to the tip of an STM and bridges the gap between the tip and the surface. In this configuration, our measurements establish a clear correlation between the zero-bias Kondo resonance and the vibrational excitations. Actually, the two side peaks in the conductance spectra can be assigned to the excitation of the stretching mode of the Mn–N_iso_ bonds of the MnPc molecule. In addition, due to the Kondo correlation, large values of the thermopower are measured across the molecule junction. We therefore conclude that single molecules that bear a Kondo impurity are interesting in connection with thermopower applications. Ultimately, a better understanding of the interactions of the molecules with their environment has been achieved.

## Experimental

Experiments were performed in a UHV system with a base pressure *p* < 10^−10^ mbar, equipped with a low-temperature scanning tunneling microscope (Modified Createc LT-STM) equipped with a vector magnetic field of 1 T. As described in [21], the Au(111) single crystal was cleaned by repeated cycles of Ne^+^ ion bombardment followed by thermal annealing at 800 K. The MnPc molecules were evaporated from an Al_2_O_3_ crucible heated by means of a tantalum filament. Before the deposition, the MnPc powder (purity > 95%) was degassed for several hours. Molecules have been deposited at a rate of 0.2 ML/min at an evaporation temperature of 250 °C on the gold substrate kept at room temperature. The sample is then transferred into the STM chamber. All STM/STS measurements have been performed at *T* = 4.5 K. Tungsten tips were flash-annealed in UHV and conditioned in situ by indentation into the Au substrate. STM images were acquired in constant-current mode, while the bias voltage is applied to the sample with respect to the tip kept at a virtual ground. The d*I*/d*V*(*V*) signal was measured by a lock-in technique, applying a modulation voltage of 1 mV (rms) at a frequency of 900 Hz.

For the thermopower experiment, the sample temperature is controlled by means of an embedded Zener diode heater, whereas the tip is thermally connected to the LHe cryostat. The temperature is measured within ±0.1 K by means of a thermocouple on the sample. The thermopower measurement is a delicate issue and the relevance of our approach is mainly tested a posteriori by two means: (i) We noticed that a long waiting time prevents the Δ*T* to be maintained constant for more than an hour or so. For this reason, the d*I*/d*V* measurement is carried out in a short period of time (few minutes) at each temperature during the dynamic sample-temperature increase. (ii) The data could not be explained by other means as the thermopower effect, in particular, the Kondo theory alone (Kondo broadening with temperature) is unable to explain the changes as shown in section “Parameter-dependent transport in a molecular junction”.
